# Comparison of Patients’ Perceived Quality of Primary Care Between Urban and Rural Community Health Centers in Guangdong, China

**DOI:** 10.3390/ijerph17134898

**Published:** 2020-07-07

**Authors:** Aiyun Chen, Shanshan Feng, Liang Zhang, Leiyu Shi

**Affiliations:** 1 School of Health Management, Guangzhou Medical University, Guangzhou 511436, China; chenaiyun@gzhmu.edu.cn (A.C.); fengshsh@gzhmu.edu.cn (S.F.); 2 Health Policy and Management, School of Public Health, John Hopkins University, Baltimore, MD 21205, USA; 3 Research Centre of Rural Healthcare Services, School of Medicine and Health Management, Tongji Medical College, Huazhong University of Science and Technology, Wuhan 430030, China; zhangliang@mails.tjmu.edu.cn

**Keywords:** primary care, community health centers/stations, township health center, rural health station, quality of care

## Abstract

Background: A series of reforms were implemented to improve the quality of primary care services in China. This study aims to assess patients’ perceived quality of primary healthcare between rural and urban community health centers in Guangdong. Methods: A cross-sectional survey was conducted from July to December 2015 in Guangdong. We surveyed 1010 respondents who visited either community health centers/stations (CHCs/CHSs) in urban areas or township health centers/rural health stations (THCs/RHSs) in rural areas. A validated Chinese version of the Primary Care Assessment Tool-Adult Short Version (PCAT-AS), representing ten primary care domains, was used to collect information on patients’ primary care experiences. A *t*-test was used for comparison on domain scores and total scores between patients from CHCs/CHSs and THCs/RHSs. An analysis of covariance was employed to compare the adjusted PCAT domain scores and total scores. Multilevel models were used to explore factors associated with PCAT total scores. Results: Overall, patients reported a lower level of experience of community orientation and family centeredness compared to other primary care domains. Patients from THCs/RHSs settings in the rural area reported better primary care experience in four domains, including first contact, accessibility, ongoing care, and community orientation. Higher education background and those with a chronic disease were associated with better primary care experience, after controlling for confounding factors. Patients who preferred primary care institutions when getting sick or used health services more frequently reported better primary care experiences. Conclusion: Continued efforts are needed to strengthen primary care performances, particularly in a community orientation and family centeredness. Primary care delivery in CHCs/CHSs settings should be improved in four domains, including first contact, accessibility, ongoing care, and community orientation.

## 1. Introduction

Achieving universal health coverage is an important health goal for all nations. It means individuals, regardless of gender, economic status, disease status, or healthcare delivery system experienced, should be provided equitable access to basic health services [1]. In 2008, the World Health Organization (WHO) urged that primary care be used as a model to provide fair and efficient care and primary care systems be strengthened in all countries [2]. Although the services and models of primary care in different countries are substantially influenced by national context and culture, a consensus has been achieved on the function of primary care. The primary care is considered to contribute to better health outcomes and lower healthcare costs, as well as have the potential to reduce health disparities [3,4,5,6,7,8,9]. The World Health Organization (WHO), the National Institutes of Health (NIH), and many experts have defined the characteristics of primary care as accessibility, first contact, comprehensiveness, continuity, and coordination. Furthermore, it has been widely accepted by experts that the participants’ self-reported perspective is a reliable means to assess the quality of primary care [4,6,8,9].

As socioeconomic conditions and healthcare development vary in urban and rural areas in China, the healthcare delivery systems implemented in the city and rural areas are different. In rural areas, there is a three-tiered healthcare delivery system, with rural health stations (RHSs) at the bottom, township health centers (THSs) in the middle, and country hospitals at the top. In urban areas, the healthcare delivery system consists of two levels, including community health centers/stations (CHCs/CHSs) and hospitals. Both CHCs/CHSs in urban areas and THCs/RHSs in rural areas are now mostly financed by government subsidies to provide primary care, such as chronic disease management and general practitioner services. However, no restrictions on medical institutions are made for seeking primary care services in both urban and rural areas. In rural areas, THCs and RHSs are the main primary care institutions, and the outpatient departments of county hospitals also provide primary care services.

Meanwhile, more and more residents bypass CHCs to seek primary care in tertiary hospitals in the urban areas [10,11,12]. The most common reason for patients’ not choosing primary health facilities is their doubts about their service quality [13,14]. However, seeking primary care in hospitals has several adverse consequences, such as a lower accessibility due to long distances and waiting times, weakened continuity because of limited patient-doctor contact, and higher healthcare expenditures [13,15].

Aiming to address the problems caused by seeking hospital care for all health problems, the Chinese government has implemented a series of reforms, such as investing in infrastructure and general practitioner training, as well as the Zero Markup for Essential Medicine Program (the policy that terminated the economic incentives behind prescriptions by forbidding healthcare institutions from claiming, at most, a 15 percent markup of medicines and providing subsidies to compensate healthcare institutions’ reductions in revenues), to improve the quality and accessibility of primary care services. However, the patient’s utilization of primary care services is still much lower than what policymakers hoped for [16]. Moreover, neither a health insurance policy nor the gatekeeper policy can restrict patients to seek primary care in primary care institutions. To address this predicament, improving the quality of primary healthcare delivered by primary care facilities is the best solution.

We did the literature search to obtain related evidence on patients’ perceived quality of primary healthcare. Studies from the United States, Brazil, Canada, Argentina, South Africa, Japan, and other locations worldwide evaluated the quality of primary care in different healthcare models [14,17,18,19,20,21,22,23,24,25,26,27,28]. As for China, some studies compared the patients’ perceived quality of primary care provided in primary healthcare, secondary, and tertiary settings [15,29,30,31], but no studies have been conducted to compare the patients’ perceived quality of primary care in urban areas and rural areas in Guangdong and other similar areas in China.

This study aims to assess patients’ perceived primary healthcare experiences in primary care facility settings between THCs/RHSs in rural areas and CHCs/CHSs in urban areas in Guangdong Province. Results of the study would provide input for policymakers to improve primary care performances and reduce the gap between urban and rural settings. 

The remainder of the paper is organized as follows. The next section outlines our methodology. We subsequently present and discuss our empirical findings and, finally, provide concluding remarks.

## 2. Methods

### 2.1. Study Site

Guangdong is an economically vibrant coastal province in Southern China, with the largest provincial population in China totaling 111,690,000 at the end of 2017. There are 78,020,000 of the population in cities, while 33,670,000 live in rural areas. With rapid economic development, Guangdong Province plays a leading role in the implementation of health policy initiatives aimed at enhancing primary care.

### 2.2. Study Design and Participants

**Figure 1:**
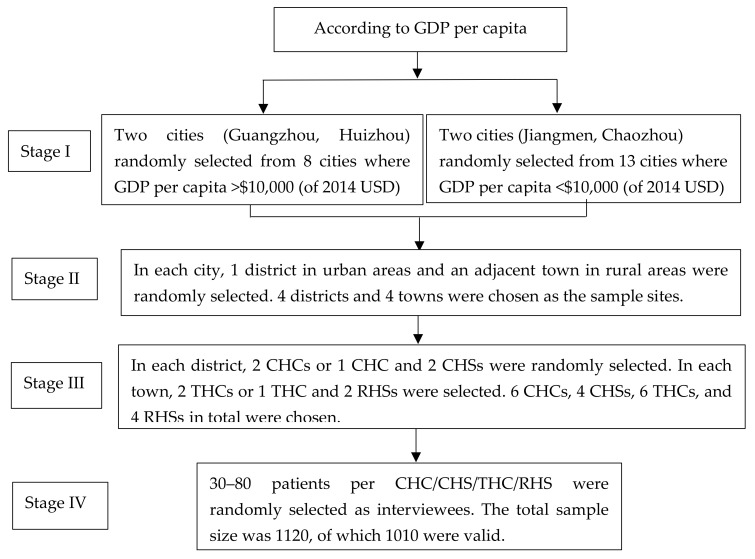
The flow chart of the sampling. The city is a geographical and administrative concept, which consists of several urban areas and rural areas. CHC = community health center, CHS = community health station, THC = township health center, and RHS = rural health station. The number of cities whose GDP (gross domestic product) per capita >10,000 was 8 in 2014, but the population covered was nearly 46% of the whole province. Therefore, 2 cities of GDP per capita >10,000, as well as 2 cities of GDP per capita <10,000, were selected.

This study was a cross-sectional survey conducted from July to December 2015 in Guangdong, China. The minimum sample size of this study was estimated as 900, with a 99% confidence interval and a power of 80% [17]. Multistage stratified sampling was adopted for this study. In the first stage, 21 cities in Guangdong Province were divided into two groups according to per capita GDP (gross domestic product), above $10,000 USD in 2014 and below $10,000. In each group, we randomly selected two cities. In the second stage, we stratified between rural and urban areas within each city. We randomly chose a district in the urban areas and an adjacent town in the rural areas in each city. In the rural areas, we enrolled patients in township health centers (THCs) and rural health stations (RHSs), while, in urban areas, we enrolled patients from community health centers/stations (CHCs/CHSs). In each district, two CHCs (or one CHC and two CHSs) were randomly chosen. In each town, two THCs (or one THC and two RHSs) were randomly chosen. In each selected facility, we set the minimum sample size at 30 and the maximum sample size at 80, and the exact number depended on the number of patients visiting when we surveyed. The study subjects were individuals aged 18 or above who ever visited the selected facility at least once. We interviewed 1120 patients with a refusal rate of approximately 10%, and the most common reason for refusal was having no time, which did not lead to sample bias (based on baseline comparisons of the two groups, i.e., participants vs. nonparticipants). The final number of included respondents was 1010, 405 from CHCs/CHSs and 605 from THCs or RHSs. Figure 1 illustrates the source of the sample.

### 2.3. Data Collection

The instrument used in our research was the Primary Care Assessment Tool, which has been widely used and tested in the United States and some other countries [3,4,8,11,15,16,17,18]. We chose the Chinese version of the Primary Care Assessment Tool-Adult Short Version (PCAT-AS, John Hopkins University, Baltimore, MD, USA) for data collection after the author’s consent. We tested the scale in our study. The reliability of the evaluation scale was assessed with Cronbach’s α, and the validity was assessed by the Kaiser-Meyer-Olkin (KMO) test. The reliability and validity were high (Cronbach’s α = 0.81 and KMO = 0.79). The PCAT-AS was developed based on the theoretical model of primary care attributes established by the John Hopkins Primary Care Policy Center and consisted of 36 items covering seven dimensions: 27 items assessing the four core dimensions of primary care first contact (with two subdomains, including first contact and accessibility); ongoing care; comprehensiveness (consisting of two subdomains, including services available and service provided); and coordination (with two subdomains, including referrals and information systems) and 9 items measuring three derivative dimensions—family centeredness, community orientation, and cultural competence. A four-point Likert-type scale was adopted as the measurement scale, where 1 = definitely not, 2 = probably not, 3 = probably, and 4 = definitely. An additional option of do not know/not sure was added in case of a lack of knowledge of a certain item, which was assigned value 2 when conducting the analyses.

The survey also covered information including respondents’ sociodemographic characteristics and health status. Variables were selected based on the widely used Andersen Behavioral Model of Health Services Utilization. According to this framework, healthcare use is influenced by individual factors consist of predisposing, enabling, and need. Predisposing factors include age, gender, education, marital status, and the other demographics. Enabling factors denote the availability of healthcare services, such as health insurance, family income, and distance to the nearest healthcare institutions. Need factors take health status into account by general health status and other chronic health conditions. Furthermore, considering that patients’ preferences and utilization of primary care institutions will affect the evaluation of services, we also included whether PHC is preferred and the number of visits to PHC as independent variables. All variables, as well as the coding method involved in this study, are presented in Table 1. 

Students from the School of Health Management of Guangzhou Medical University were recruited as interviewers. A one-week training was given to train the investigators in the survey skills and explain the meaning of each item in the questionnaire. 

The questionnaire was completed mainly by the respondents themselves, and items and response choices were read to participants who had difficulty in reading (because of illiteracy or poor vision). The time to complete a questionnaire was 18 to 22 min. A valid questionnaire was defined as having <5% missing data. Subjects with missing data were excluded from the final analyses. Since only less than 5% of the subjects had missing data, their exclusion did not affect either the sample size or the representativeness of the sample (based on baseline comparisons of the two groups, i.e., those with significant missing values and those without).

### 2.4. Data Analysis

The overall aim of the analysis was to compare the primary healthcare quality (represented by PCAT scores) between CHCs/CHSs in urban areas and THCs/RHSs in rural areas and factors influencing PCAT total scores in Guangdong. Data were double entered into Epidata 3.1 (The EpiData Association, Odense, Denmark) and exported to PASW Statistics 18.0 (IBM, New York, NY, USA) for analysis. PCAT scores were calculated for item scores, domain scores, and total scores, respectively. The score of each domain was the sum scores of each item within the domain. The total PCAT scores were calculated by summing the scores for the nine domains, except for the coordination (referrals) domain, since only 601 participants reported the experience of referral. Higher scores indicated better patient primary care experiences, according to the PCAT Manual.

The chi-square test was conducted to test for differences of characteristics between the respondents, accessing different types of medical institutions. The *t*-test was used for comparison on domain scores and total scores between patients from CHCs/CHSs and THCs/RHSs. An analysis of covariance (ANCOVA) was employed to compare the adjusted PCAT domain scores and total scores. Considering the possible clustering effect in stratified sampling, we applied multilevel models to explore factors associated with PCAT total scores.

### 2.5. Ethics

This study was approved by the Ethics Committee of Guangzhou Medical University (IORG No: IORG20150416) and was carried out following the principles of the Declaration of Helsinki. The study participants were informed of the purpose of the study and assured of keeping their identity and responses confidential. Written informed consent was signed before the questionnaire was administered to the participants. Refusal to participate or to discontinue participation at any time was allowed.

## 3. Results

Table 1 summarizes the characteristics of the participants. Patients accessed primary care in CHCs/CHSs in urban areas or THCs/RHSs in rural areas and differed in gender, age, education background, income, self-reported health status, the time it takes to walk to the nearest primary care institution, whether patients used primary care institutions as the first choice when getting sick, and the number of visits to a primary care institution the previous year. The CHCs/CHSs users were more likely to be female, aged 65 years and above, graduated from middle school, and had family monthly incomes under $1157 (of 2014 USD). THCs/RHSs users were more likely to take less than 30 min walking to the nearest primary care institution and preferred to use THCs/RHSs when getting sick. 

Table 2 presents the results of the comparison of the PCAT total scores and domain scores between patients in urban and rural areas. After adjustments were made, the means of the PCAT total scores were 70.43 among all participants. The top three domain scores after standardization were cultural competence, comprehensiveness (services provided), and first contact. The scores were 9.74, 9.43, and 6.98, respectively. The three domains with the lowest scores after standardization were community orientation, family centeredness, and accessibility. Means of the three domain scores were 5.26, 5.47, and 7.68, respectively.

**Figure 2:**
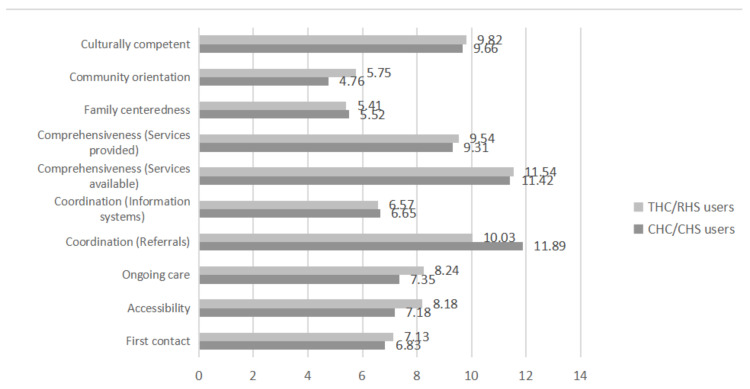
Comparisons of Primary Care Assessment Tool (PCAT) scores in each domain between the two groups.

After controlling for confounding factors, including sociodemographic characteristics, health status, medical expenditures, and the utilization of primary care services, the means of the PCAT total scores reported by THCs/RHSs respondents were higher than those from CHCs/CHSs users in urban areas; the scores were 72.17 and 68.69, respectively. In addition, the means of the first contact, accessibility, ongoing care, and community orientation in THCs/RHSs respondents in rural areas were significantly higher than those from CHCs/CHSs users in urban areas. Nevertheless, the means of coordination (referrals) reported by CHCs/CHSs users in urban areas were higher than THCs/RHSs users in rural areas. Comparisons between the two groups on the ten domains are shown in Figure 2.

The linear mixed models show that the medical institution type, education background, chronic diseases, preferred medical institution, and the number of visits to a primary care institution were significantly associated with the PCAT total scores. Considering that there are many independent variables, and there may be interactions, we applied the variance inflation factor (VIF) to test the multicollinearity. The results showed that all VIF values were less than 5, suggesting that no significant collinearity existed. In models I–IV, after controlling for various characteristics of the respondents, the respondents whose education was college and above had significantly lower PCAT total scores when compared with those who had primary school and below education backgrounds. Respondents without chronic diseases had lower PCAT total scores than those who had chronic diseases. Furthermore, compared to those who preferred THCs/RHSs when getting sick, participants who went to secondary and above hospitals had lower PCAT total scores. Patients who visited primary care institutions more than seven times reported higher PCAT total scores than those whose number of visits to primary care institution was less than three times. Details are presented in Table 3.

## 4. Discussion

This study aimed to compare patients’ perceived quality of primary healthcare delivered by CHCs/CHSs in urban areas and THCs/RHSs in rural areas in China. The study added evidence that community-based primary care institutions could perform well in cultural competence, comprehensiveness (services provided), and first contact domains. However, the two domains of community orientation and family centeredness did not receive high evaluations.

Cultural competence received the highest score in all the domains, indicating patients would recommend familiar doctors to their relatives, and the doctors in the primary care institutions can speak both Mandarin and Cantonese and provide traditional Chinese medicine to patients in need. Since both Mandarin and Cantonese are the most common languages spoken in Guangdong, medical staff, especially those working in primary care facilities, are required to speak Cantonese, in addition to Mandarin. Traditional Chinese medicine has a long tradition in use in Guangdong, which has been promoted in grassroots medical institutions and widely accepted by residents. 

THCs/RHSs received higher PCAT total scores than CHCs/CHSs, even after controlling for patients’ demographic characteristics, health status, and health service utilization, suggesting that THCs/RHSs in rural areas provide higher levels of primary care compared to the CHCs/CHSs settings in urban areas. Primary care delivery in THCs/RHSs settings performed better in four domains, including first contact, accessibility, ongoing care, and community orientation. 

The results of well-performed first contact and accessibility in THCs/RHSs may be explained by the following factors: convenient travel distance to THCs/RHSs, patients and doctors were familiar to each other, and doctors were not limited to workdays. The higher performance in ongoing care suggested patients would keep a closer relationship with doctors, such as seeing the same doctor every time, and called doctors for medical consultations. These findings are consistent with previous research [31]. Compared to CHCs/CHSs in urban areas, THCs/RHSs in rural areas are smaller medical institutions; the provider and the patient are more willing to build a long-term relationship to foster mutual understanding and a knowledge of the other’s expectations and needs.

However, CHCs/CHSs in urban areas performed better in coordination (referrals), which means doctors in CHCs/CHSs were likely to advise their patients to seek medical service in the upper-level hospitals and provide a referrals service to the patients in need. This could be due to the health insurance reimbursement stipulation. Since 2014, some cities in Guangdong implemented a preferential policy that patients referred from CHCs/CHSs received an extra 10 or 15 percent reimbursement for healthcare expenditures occurring at hospitals.

Consistent with findings from the previous studies [30,31], the standardized mean score for community orientation was the lowest among all domains, which suggested that community-based health services were not well-performed. Community-oriented primary care requires meeting the healthcare needs of not only the patients and families but, also, residents in the community [32]. Though scores of THCs/RHSs were significantly higher when compared with CHCs/CHSs, scores in this domain barely met the minimum expectation and should be further improved.

Family-centeredness was the other domain not receiving a satisfying evaluation in THCs/RHSs, as well as CHCs/CHSs. It suggested that doctors would not seek advice from patients or their family members when making treatment plans. This finding was in-line with several previous studies [33,34,35]. Though many experts called for more autonomy and participation for patients, it was difficult for patients to achieve equality in their medical decisions due to insufficient medical knowledge or communication barriers [13,36]. This situation usually occurred not only in primary care institutions but, also, in hospitals.

The results indicated that education background was the only demographic character affecting patients’ experiences of primary care when controlling for confounding factors. Compared to those with primary school and below education, those who had a college degree and above reported lower primary care scores. This may be explained by the fact that patients with high education levels have more diversified access to medical services and higher expectations for the quality of medical service.

Consistent with other studies [37,38], having chronic diseases was associated with better primary care quality, after controlling for other influencing factors. Further subanalyses showed that better coordination in terms of referrals and the information system and comprehensiveness (services provided) may account for the higher PCAT scores among those who had a chronic disease. Coordination in terms of the information system can be explained by the health record system and regular follow-up services for patients with chronic diseases due to an effective implementation of the basic public health service package in 2009, which was funded by central or local governments. This finding was consistent with the conclusion that patients with chronic conditions reported better coordination in terms of an information system [37]. A better coordination in terms of referrals may be due to the implementation of a general practitioner system since 2014 in Guangdong, which aimed to deliver integrated healthcare, including referrals for patients with chronic diseases. Furthermore, this study showed that primary care institutions performed well in providing comprehensiveness (services provided) services for patients with chronic diseases, which was important in dealing with the mounting challenge of addressing noncommunicable diseases.

Compared to those who preferred hospitals, patients who preferred primary care institutions when getting sick rated higher scores on their primary care experiences. There were significant differences in eight domains between the two groups, except for coordination and family-centeredness. This may be explained by the fact that patients who preferred primary care institutions tended to be more satisfied with primary care facilities, and patients who were more satisfied with primary care facilities also gave higher scores for the quality of their primary care experiences. The conclusion that higher patient satisfaction was associated with higher PCAT total scores was corroborated by several previous studies [15,29].

More health service utilization was positively associated with a better assessment of primary care quality after controlling for confounding factors. Patients who visited primary health institutions seven times or more in the previous year gave a higher score. This finding is in-line with a previous study in Guangdong, which reported that those who visited medical facilities with a higher frequency tended to report better primary care experiences [29]. Further subanalyses showed that better ongoing care, better coordination in terms of an information system, superior comprehensiveness (services provided), and better cultural competence might account for the higher PCAT scores among those who made more use of primary care services. Furthermore, the patients who visited medical facilities with a higher frequency are mainly those in chronic conditions or the senior citizens, who were included in the basic public health service package that allowed them access to more primary care services.

Consistent with a previous study, the presence of health insurance was not associated with the PCAT total scores [31]. Respondents’ sociodemographic characteristics, such as gender, age, and family monthly income, did not influence the perceived quality of primary care services, which was inconsistent with previous studies [15,29,38].

There were several limitations in the study. First, some unmeasured confounders could have led to a potential residual confounding of the data. Second, we conducted a cross-sectional survey from July to December. The patients’ disease patterns we met may be different from those collected in other seasons, which may affect the evaluation of their perceived service quality. Furthermore, the impact of time should be considered, especially with the implementation of a series of reforms aimed at strengthening community-based primary care delivery systems. Further studies about whether the patients’ perceived quality of primary medical services have improved should be conducted. Third, the survey data were based entirely on self-reports, and recall bias could be a potential limitation that reduces the reliability in our analysis. Fourth, this study examined patients’ perceived experiences. Differences in patients’ characteristics may influence their assessment of primary care services. Further research is needed to investigate providers to verify the consistency of the demand side and provider side.

## 5. Conclusions

Despite these limitations, this study provides new evidence in comparing primary care quality between THCs/RHSs in the rural areas and CHCs/CHSs in the urban areas of China. The results of the study show that community-based primary care institutions performed well in cultural competence, comprehensiveness (services provided), and first contact domains. However, two domains, including community orientation and family centeredness, did not meet patients’ expectations. Additionally, primary care delivery in THCs/RHSs settings had better overall performances and performed better in four domains, including first contact, accessibility, ongoing care, and community orientation. These findings will be significant in directing policymakers, improving the quality of primary healthcare and reducing health inequity in patients between cities and the countryside. At first, continued efforts are needed to strengthen community orientation and family centeredness. For instance, primary healthcare institutions providing more traditional Chinese medicine (TCM) that fits with patients’ needs and doctors involving patients and their families in medical decision-making. Furthermore, a relatively long-term and stable relationship between doctors and patients can be built through the patient-GP (general practitioner) contract service, and it will improve the first contact, accessibility, and ongoing care of primary care delivery in CHCs/CHSs settings.

## Figures and Tables

**Table 1:** Comparison of characteristics between respondents accessing primary care in CHCs/CHSs and THCs/RHSs.

Characteristics	All Participants *n* = 1010 (100.0)	CHC/CHS *n* = 405 (%)	THC/RHS *n* = 605 (%)	χ^2^	*p*-Value
Gender					
Male	412 (40.79)	134 (33.09)	278 (45.95)	16.62	<0.001
Female	598 (59.21)	271 (66.91)	327 (54.05)		
Age					
18–34	72 (7.13)	22 (5.43)	50 (8.26)	13.07	0.011
35–49	123 (12.18)	36 (8.89)	87 (14.38)		
50–64	336 (33.27)	137 (33.83)	199 (32.89)		
≥65	479 (47.42)	210 (51.85)	269 (44.47)		
Marital status					
Married	840 (83.17)	328 (80.99)	512 (84.63)	2.30	0.130
Unmarried (single/divorced/windowed)	170 (16.83)	77 (19.01)	93 (15.37)		
Education background					
Primary school and below	492 (48.71)	148 (36.54)	344 (56.86)	42.81	<0.001
Middle school	433 (42.87)	208 (51.36)	225 (37.19)		
College degree and above	85 (8.42)	49 (12.10)	36 (5.95)		
The income of family monthly (of 2014 USD)					
<$495.9	706 (69.90)	208 (51.36)	498 (82.31)	112.99	<0.001
$495.9–$1157.0	253 (25.05)	169 (41.73)	84 (13.88)		
≥$1157.0	51 (5.05)	28 (6.91)	23 (3.81)		
Medical insurance					
Have	988 (97.82)	397 (98.02)	591 (97.69)	3.24	0.293
No	22 (2.18)	8 (1.98)	14 (2.31)		
The proportion of medical expenditure to total family expenditures					
<30%	853 (84.46)	334 (82.47)	519 (85.78)	3.62	0.308
≥30%	157 (15.54)	71 (17.53)	86 (14.22)		
Self-reported health status					
Well	374 (37.03)	126 (31.11)	248 (40.99)	10.91	0.004
General	454 (44.95)	204 (50.37)	250 (41.32)		
Bad	182 (18.02)	75 (18.52)	107 (17.69)		
Chronic conditions					
Yes	462 (45.74)	200 (49.38)	262 (43.31)	6.89	0.122
No	548 (54.26)	205 (50.62)	343 (56.69)		
Walking to the nearest primary care institutions (minutes)					
<30	858 (84.95)	326 (80.49)	532 (87.93)	10.50	0.001
≥30	152 (15.05)	79 (19.51)	73 (12.07)		
Preferred primary care institutions					
Yes	696 (68.91)	143 (35.31)	553 (91.40)	180.82	<0.001
No	314 (31.09)	262 (64.69)	52 (8.60)		
Number of visiting primary care institutions					
≤3	428 (42.38)	146 (36.05)	282 (46.61)	14.79	0.001
4–6	218 (21.58)	86 (21.23)	132 (21.82)		
≥7	364 (36.04)	173 (42.72)	191 (31.57)		

CHC = community health center, CHS = community health station, THC = township health center, and RHS = rural health station. The proportion of medical expenditure to total family expenditures means out-of-pocket payments to total family expenditures (including food, education, housing, and other expenditures) in the previous year.

**Table 2:** Comparison of the primary care assessment scores in CHC/CHS and THC/RHS.

Domain	All participants (*n* = 1010)		CHC/CHS (*n* = 405)		THC/RHS (*n* = 605)		*t*-Value	*p*-Value
	Mean (SE)	Standardized Score	Mean (SE)	Standardized Score	Mean (SE)	Standardized Score		
Unadjusted								
First contact **	7.01 (0.06)	7.79	6.78 (0.98)	7.53	7.16 (0.73)	7.96	3.13	0.002
Accessibility **	7.77 (0.09)	6.48	6.96 (0.14)	5.80	8.32 (0.12)	6.93	7.42	<0.001
Ongoing care **	7.89 (0.09)	6.58	7.24 (0.14)	6.03	8.31 (0.11)	6.93	6.08	<0.001
Coordination (Referrals) **	10.82 (0.38)	7.21	11.76 (0.61)	7.84	10.13 (0.47)	6.75	−2.24	0.026
Coordination (Information systems) **	6.60 (0.07)	7.33	6.83 (0.13)	7.59	6.44 (0.09)	7.16	−2.60	0.009
Comprehensiveness (Services available)	11.49 (0.09)	7.73	11.33 (0.16)	7.55	11.60 (0.12)	7.73	1.40	0.163
Comprehensiveness (Services provided)	9.45 (0.10)	7.88	9.36 (0.15)	7.80	9.51 (0.12)	7.93	0.76	0.451
Family centeredness	5.45 (0.08)	6.06	5.47 (0.12)	6.08	5.44 (0.09)	6.04	−0.21	0.833
Community orientation **	5.35 (0.07)	5.94	4.69 (0.10)	5.21	5.80 (0.09)	6.44	7.85	<0.001
Culturally competent	9.75 (0.07)	8.13	9.63 (0.11)	8.03	9.83 (0.09)	8.19	1.43	0.152
Total scores **	70.70 (0.47)	73.65	68.32 (0.73)	71.17	72.41 (0.59)	75.43	4.37	<0.001
Adjusted								
First contact **	6.98 (0.06)	7.76	6.83 (0.09)	7.59	7.13 (0.08)	7.92	5.47	0.024
Accessibility **	7.68 (0.09)	6.40	7.18 (0.15)	5.98	8.18 (0.12)	6.82	25.56	<0.001
Ongoing care **	7.80 (0.09)	6.50	7.35 (0.14)	6.13	8.24 (0.11)	6.87	22.99	<0.001
Coordination (Referrals) **	10.91 (0.38)	7.27	11.89 (0.59)	7.93	10.03 (0.53)	6.69	5.71	0.017
Coordination (Information systems)	6.61 (0.08)	7.34	6.65 (0.12)	7.39	6.57 (0.10)	7.30	0.26	0.608
Comprehensiveness (Services available)	11.48 (0.09)	7.65	11.42 (0.15)	7.61	11.54 (0.12)	7.69	0.30	0.587
Comprehensiveness (Services provided)	9.43 (0.10)	7.86	9.31 (0.16)	7.76	9.54 (0.13)	7.95	1.22	0.271
Family centeredness	5.47 (0.08)	6.08	5.52 (0.12)	6.13	5.41 (0.10)	6.01	0.48	0.493
Community orientation **	5.26 (0.07)	5.84	4.76 (0.12)	5.29	5.75 (0.09)	6.39	40.78	<0.001
Culturally competent	6.74 (0.07)	7.49	6.74 (0.11)	7.49	6.74 (0.09)	7.49	0.16	0.983
Total scores **	67.76 (0.45)	70.58	65.43 (0.71)	68.16	69.32 (0.58)	72.21	18.14	0.001

CHC = community health center, CHS = community health station, THC = township health center, RHS = rural health station, and SE = standard error. Six-hundred and one respondents reported having an experience of referral, including 272 from CHC/CHS and 329 from THC/RHS. The total scores were calculated by summing the mean scores for 9 domains, except for the coordination (referrals) domain. The standardized score of every domain was the means of the item score divided by the item total score × 10, aiming to compare the level of domains. The *t*-test was carried out for unadjusted domain scores, and the analysis of covariance (ANCOVA) was carried out for adjusted domain scores, which were adjusted for gender, age, marital status, education background, family monthly income, proportion of medical expenditure to total family expenditures, self-reported health status, chronic conditions, walking to the nearest primary care institutions, preferred primary care institutions, and number of visiting primary care institutions. ** means there was a difference in PCAT scores in this domain.

**Table 3:** Linear mixed model results on primary care assessment total scores.

Variables	Model I		Model II		Model III		Model IV	
	β (95% CI)	*p*-Value	β (95% CI)	*p*-Value	β (95% CI)	*p*-Value	β (95% CI)	*p*-Value
Medical institution type								
THC/RHS (ref)								
CHC/CHS	−2.17 (−3.34, −0.61)	<0.001	−1.19 (−2.91, −0.11)	0.005	−2.55 (−4.96, −1.23)	<0.001	−3.58 (−7.69, −1.12)	<0.001
Gender								
Male (ref)								
Female			−0.23 (−1.08, 0.71)	0.783	−0.25 (−1.10, 0.60)	0.798	0.46 (−0.37, 1.18)	0.650
Age								
18–34 (ref)								
35–49			0.17 (−0.52, 0.76)	0.877	0.99 (−0.60, 2.58)	0.613	1.21 (−0.32, 2.88)	0.332
50–64			−1.02 (−3.18, 1.71)	0.582	−1.41 (−2.24, 0.53)	0.365	−0.86 (−3.07, 1.87)	0.814
≥65			−0.05 (−0.67, 0.81)	0.886	−0.38 (−1.55, 1.45)	0.756	−0.38 (−1.92, 1.39)	0.806
Marital status								
Married (ref)								
Unmarried (single/divorced/windowed)			−1.77 (−4.52, −0.21)	0.063	−2.77 (−5.22, −0.23)	0.076	−1.49 (−3.85, 0.86)	0.208
Education background								
Primary school and below (ref)								
Middle school			−1.32 (−3.22, 0.51)	0.122	−1.34 (−3.23, 0.52)	0.133	−0.46 (−1.77, 2.39)	0.206
College degree and above			−6.61 (−10.07, −1.27)	0.001	−5.45 (−9.12, −2.44)	0.001	−3.89 (−8.51, 0.71)	0.001
The income of family monthly (of 2014 USD)								
<$495.9 (ref)								
$495.9–$1157.0			−0.81 (−1.97, 1.12)	0.383	−1.28 (−3.25, 0.91)	0.208	−1.10 (−3.13, 0.97)	0.290
≥$1157.0			−2.41 (−5.23, −1.03)	0.065	−2.76 (−4.98, −1.34)	0.178	−2.88 (−6.68, 1.53)	0.353
Self-reported health status								
Well (ref)								
General					−0.09 (−0.53, 0.46)	0.893	−0.21 (−1.92, 0.76)	0.488
Bad					−2.46 (−5.24, 0.11)	0.071	−2.11 (−5.78, −0.15)	0.191
Chronic conditions								
Yes (ref)								
No					−4.61 (−9.23, −1.24)	<0.001	−3.52 (−5.32, −1.69)	<0.001
Medical insurance								
Have (ref)								
No					−2.16 (−4.53, 1.21)	0.657	−2.87 (−6.33, 1.42)	0.511
The proportion of medical expenditure to total family expenditures								
<30% (ref)								
≥30%					0.37 (−0.07, 1.54)	0.689	0.92 (−0.38, 2.32)	0.564
Walking to the nearest primary care institutions (minutes)								
<30 (ref)								
≥30							−0.59 (−1.07, 0.89)	0.643
Preferred primary care institutions								
Yes (ref)								
No							−6.64 (−9.76, −3.52)	<0.001
Number of visiting primary care institutions								
≤3 (ref)								
4–6							1.55 (−0.57, 3.66)	0.1524
≥7							4.01 (1.66, 6.34)	0.002

Considering the possible clustering effect in stratified sampling, we apply multilevel models in the analysis. It means that we set patients as low-level units and medical institutions as high-level units and use linear mixed models. Model I: Included only medical institution types. Model II: Controlled for sociodemographic characteristics. Model III: Controlled for sociodemographic characteristics and health status. Model IV: Controlled for sociodemographic characteristics, health status, and the utilization of primary healthcare. CHC = community health center, CHS = community health station, THC = township health center, and RHS = rural health station. CI: confidence interval, Ref: reference group.

## Abbreviations

CHCs: community health centers, CHSs: community health stations, THCs: township health centers, RHSs: rural health stations, SE: standard error,GDP: gross domestic product, CI: confidence interval, Ref: reference group, ANCOVA: analysis of covariance, and GP: general practitioner.
